# Phylogeography of plastid DNA sequences suggests post-glacial southward demographic expansion and the existence of several glacial refugia for *Araucaria angustifolia*

**DOI:** 10.1038/s41598-019-39308-w

**Published:** 2019-02-26

**Authors:** Valdir Marcos Stefenon, Gustavo Klabunde, Rafael Plá Matielo Lemos, Marcelo Rogalski, Rubens Onofre Nodari

**Affiliations:** 1 Nucleus of Molecular Ecology and Plant Micropropagation, Universidade Federal do Pampa, Campus São Gabriel, Av. Antonio Trilha 1847, 97300-162 São Gabriel, RS Brazil; 2 Graduate Program on Plant Genetic Resources, Universidade Federal de Santa Catarina, 88040-900 Florianópolis, SC Brazil; 3 Laboratory of Plant Molecular Physiology, Department of Plant Biology, Universidade Federal de Viçosa, 36570-900 Viçosa, MG Brazil

## Abstract

Despite the high diversity of the Southern South American environments, the patterns and processes driving both their species diversity and demographic history are still poorly known and are a challenging task. In this study, we evaluate plastid DNA sequences of the conifer species *Araucaria angustifolia* aiming to (i) assess the species genetic structure within its main range of occurrence, (ii) infer its population demographic history, looking for evidence of southward expansion, (iii) search for evidence of glacial refugia within the species distribution area and (iv) discuss some conservation and management strategies for this species. Twenty haplotypes were identified, revealing the presence of three distinct genetic groups across the geographic range of the species and structuring the populations into Northern, Central and Southern groups. Our results suggest the occurrence of post-glacial expansion of *A. angustifolia* towards the south, as well as the existence of at least three refugia within the species occurrence area. Testing the occurrence of historical demographic expansion, we suggest that genetic groups experienced fluctuations in effective size, associated to a structured distribution of populations. The identification of three genetic groups in this study corroborates the proposition of using the geographic distribution of *A. angustifolia* for selecting *in situ* conservation areas, for planning seed collection for *ex situ* conservation, as well as for the delineation of seed zones.

## Introduction

The Atlantic Forest has remarkable biological diversity and a variety of vegetation types, including montane grassland, mangrove, restinga, tropical forest and subtropical forest, within a single Brazilian biome. Because habitats are often megadiverse^1,2^ and only a small portion of this species multiplicity has been studied in an evolutionary context, the reconstruction of evolutionary processes involved in the diversification in these regions is a challenging task^3^.

Quaternary climatic shifts altered the spatial distribution of species and environments worldwide, acting as a main diversification driver in both temperate and tropical regions^4,5^. During the Pleistocene glaciations, many species moved towards lower latitudes, whereas other species migrated to refugia, which fragmented a once relatively contiguous distribution into multiple patchily distributed populations^6^. According to a paleomodel proposed by Carnaval & Moritz^7^, in the Atlantic Forest these refugia were restricted to the northern limits of the biome, mainly in coastal areas, while severe forest contractions nearly eliminated forests at higher latitudes. The southern Atlantic Forest would subsequently have been colonized from refugia in the north during the Holocene^3^, through southward expansion. However, a most recent study on the environmental drivers of diversity in Brazilian subtropical highlands^8^ suggests the existence of areas suitable for forest species occurrence at higher southern latitudes during the Last Glacial Maximum.

As well, the phylogeographic pattern of genetic structuring previously described for *Araucaria angustifolia* populations^9–12^ and the latitudinal discontinuous distribution of the forests with araucaria suggest, in addition to southward expansion, the existence of further refugia at higher southern latitudes. Several phylogeographic studies in South America have identified multiple refugia^13^, even at high latitudes, as evidenced by plastid DNA markers of *Nothofagus nervosa*^14^.

Despite the diversity richness and importance of the Atlantic Forest, as well as the advances in methodological and statistical analyses, the patterns and processes driving its species diversity are still poorly known^13^, particularly in the southernmost portion. The southern Atlantic Forest has a subtropical climate, mainly in the highlands, and holds predominantly montane grasslands and subtropical forests with araucaria. *Araucaria angustifolia* (Bert.) O. Ktze. is a long-lived dioecious conifer species endemic to the subtropical Atlantic Forest in the highlands of Brazil and small patches of forest in Argentina^15^ and Paraguay^16^. This species presents a latitudinal discontinuous distribution with a large gap dividing the occurrence area of the species in two main geographical zones in Brazil: (i) a northernmost zone that is formed by scattered medium to large forest formations at high altitudes in the Southeastern region of the country and (ii) a southernmost zone, which is composed by a much wider area originally formed by large continuous forest formations in the Southern region of Brazil. This zone includes the Argentinian and Paraguayan patches of forest with araucaria. The gap area between the northern and southern occurrence areas of *A. angustifolia* lacks register of recent population presence and only ecological simulations suggest the possibility of occurrence of the species in this area.

Previous genetic studies based on isozymes^9^ and nuclear microsatellite markers^10,11^, linked to palynological data proposed that this species has a strong phylogeographic pattern of genetic structuring that may be related to Quaternary climatic shifts and recent population expansion^17–19^. Based on the distribution of alleles and genotypes from nuclear microsatellite markers, a clear genetic structure is observed, revealing the presence of distinct genetic groups in a latitudinal pattern. Samples from the northernmost zone of the species occurrence are significantly distinguished from samples from the southern zone of occurrence. On the other hand, populations collected within the southernmost zone are less differentiated from each other. This pattern of population differentiation suggested the presence of two or three distinct genetic groups of *A. angustifolia*^10^. These outcomes allow to elaborate two main hypotheses about the current distribution of genetic groups of this species. One hypothesis concerns the southward expansion of *A. angustifolia* populations from a northern genetic group originated from a northern refuge (as proposed by Carnaval & Moritz^7^), following the retraction of the glaciated areas. Another hypothesis is related to the existence of southern glacial refugia, which originated the southernmost genetic groups, independent of the northern genetic groups. The occurrence of anthropic activities driving the geographic expansion of *A. angustifolia* within Southern Brazil was recently proposed based on archeological, genetic and ecological data^12,20^. Since these studies are based only on data from southernmost populations, without inclusion of samples representing the northern genetic group and weakly representing the most central populations in the Paraná State, those hypotheses were not tested.

Aiming to evaluate distribution patterns of genetic variation among natural populations of *A. angustifolia* across most of the species range, and to infer its demographic history, we conducted a large-scale genetic analysis based on the sequence variation of three intergenic regions of plastid DNA (ptDNA). Overall, this study aimed to test the above presented hypotheses about the current distribution of *A. angustifolia* genetic groups by exploring these ptDNA sequences. Signatures of recent demographic expansion (e.g. negative values in neutrality tests), genetic divergence among populations associated to geographic distances, and haplotype network presenting a star-like form (i.e. several haplotypes originated from a single ancestor haplotype) would suggest that the current distribution of this species can be accepted as the result of southward expansion. On the other hand, a strong genetic structure among genetic groups, and a haplotype network containing more than one putative ancestor haplotype would imply that the current distribution of *A. angustifolia* genetic groups is a consequence of the expansion of populations from different glacial refugia in the northern and in the southern zones of the species occurrence.

Following this logic and considering the results of previous studies that suggest geographic patterns of genetic differentiation in populations of *A. angustifolia* influenced by Quaternary shifts on the distribution of the genetic variants, this study intended to (i) evaluate the genetic structure of *A. angustifolia* within its range of occurrence; (ii) infer the population demographic history of this species, looking for evidence of southward expansion; (iii) search for evidence of glacial refugia within the species distribution area of *A. angustifolia*; and (iv) discuss some conservation and management strategies for this species, based on its current genetic structure and inferred evolutionary history.

## Results

### Evidence of distinct plastid genetic groups in *A. angustifolia*

The alignment of the three intergenic plastid regions generated in the present study encompasses 2,382 bp, with 16 variable sites and two indels, revealing low variation (Table 1). Twelve of these sites exhibit two variants, two sites show three variants and two sites have singleton polymorphism.

**Table 1:** Estimations of genetic diversity parameters for the pooled dataset and for each genetic group.

	Pooled dataset (n = 580)	Northern group (n = 101)	Central group (n = 195)	Southern group (n = 284)
Polymorphic sites	16	08	06	09
Indels	02	—	—	02
Number of haplotypes	20	08	06	11
Haplotypes	H1-H20	H1-H3, H10, H17-H20	H4, H9, H11-H12 and H15-H16	H4-H9 and H11-H15
Private Haplotypes	—	H1-H3, H10, H17-H20	H16	H5-H8 and H13-H14
Overall *θ*_*π*_^a^	1.947	1.644	1.539	0.848
Overall *θ*_*S*_^a^	2.306	1.542	1.026	1.283

^a^Estimations performed grouping all individuals as a single population for the whole range or for each genetic group.

**Figure 1:**
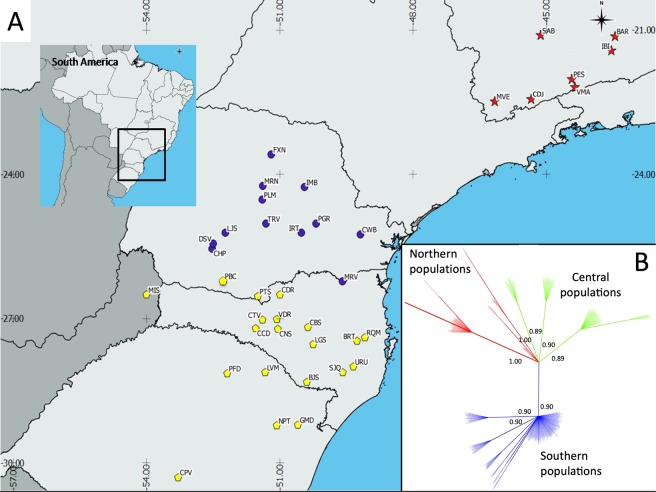
(**A**) Overview of the sampling sites within the distribution area of *A. angustifolia*. Different symbols correspond to the genetic groups identified after data analysis: red stars represent populations comprising the Northern group, blue circles represent populations comprising the Central group and yellow pentagons represent populations comprising the Southern group. (**B**) Bayesian phylogenetic tree of the 39 populations of *A. angustifolia*. Red branches correspond to individuals from populations of the Northern group; green branches correspond to individuals from populations of the Central group; blue branches correspond to individuals from populations of the Southern group.

**Figure 2:**
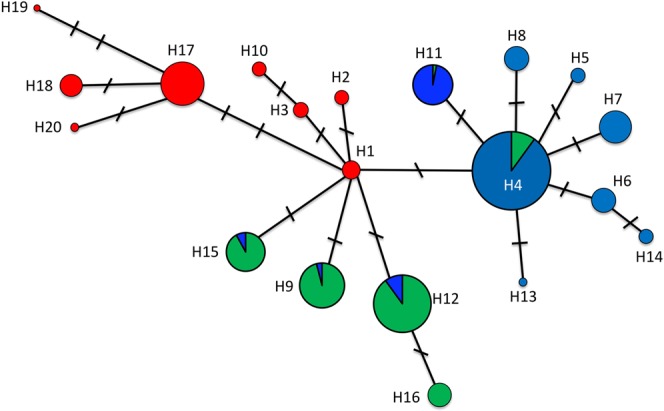
Haplotype network. Red circles are haplotypes exclusive to the North group; green circles are haplotypes exclusive to the Central group; blue circles are haplotypes exclusive to the Southern group; haplotypes shared by the Central and Southern groups represent their frequency in each genetic group with the green and blue colors representing the Central and Southern groups, respectively. The sizes of the circles correspond to the frequency of each haplotype over all populations and the length of the lines is proportional to the number of mutations.

Overall, 20 haplotypes were identified (Table 1 and Supplementary File 1). The distribution of these haplotypes corroborates previously suggested partitioning of *A. angustifolia* populations in three genetic groups based on nuclear SSR markers^10^. These three groups are replicated in the distribution of the plastid haplotypes, in the SAMOVA analysis and in the Bayesian phylogenetic inference (see below). The Northern group comprises seven populations and has eight haplotypes, all exclusive to this geographic region (Figs 1A and 2 and Supplementary File 1). The Central group is formed by 12 populations and has six haplotypes, with one haplotype exclusive to this group. The Southern group is formed by 20 populations and has 11 haplotypes, of which six are exclusive to this group (Fig. 2). No haplotype is shared with the Northern group, while five haplotypes are shared between the Central and Southern groups (Fig. 2 and Supplementary File 1). These five shared haplotypes present markedly different frequencies (Central = 0.92/Southern = 0.08, Central = 0.06/Southern = 0.94, Central = 0.96/Southern = 0.04, Central = 0.03/Southern = 0.97 and Central = 0.89/Southern = 0.11; Supplementary File 1), supporting the existence of distinct genetic groups experiencing gene flow.

In the SAMOVA analysis, the *F*_*CT*_ estimations were all significant (*p* < 0.001) and continuously increased from *K* = 2 (*F*_*CT*_ = 0.620) up to *K* = 15 (*F*_*CT*_ = 0.690) (Supplementary File 2). Such an increase in *F*_*CT*_ along with *K* is expected because of the reduction of the proportion of *F*_*SC*_-variance due to differences between populations within each group^21^. For all simulated *K* values, a group of 14 Southernmost populations (BJS, BRT, CBS, CDR, CPV, CTV, GMD, LGS, LVM, NPT, PFD, RQM, SJQ and URU) clustered together, as did a group of five Northern populations (IBI, MVE, PES, SBA and VMA). The other two northern populations (BAR and CDJ) grouped with Central populations or formed a single group, but never grouped with Southernmost populations. Estimations of *F*_*CT*_ and *F*_*ST*_ showed a pattern of stabilization after *K* = 3 (Supplementary File 2).

Bayesian phylogenetic inference of the data (Fig. 1B) subdivided populations in two main clades, one composed by Southern populations and other by Northern and Central populations originated from the same node. In a general pattern, this structure resembles the SAMOVA results for *K* = 3 (Supplementary File 2). All clades presented high posterior probability support (PP ≥ 0.89).

Considering the nuclear SSR data^10^, the haplotype distribution (Supplementary File 1), the SAMOVA analysis (Supplementary File 1) and the Bayesian phylogenetic inference (Fig. 1B), we adopt hereafter a geographical-genetic partitioning of populations in three groups: a Northern group (formed by populations from the northernmost occurrence area), a Southern group (composed by populations from the southernmost occurrence area) and a Central group, geographically intermediate between northernmost and southernmost populations, but genetically distinct from both Northern and Southern genetic groups.

### Levels of genetic diversity and structure of genetic groups

The estimations of genetic diversity were *θ*_*π*_ = 1.947 and *θ*_*S*_ = 2.306 for the pooled dataset, *θ*_*π*_ = 1.644 and *θ*_*S*_ = 1.542 for the Northern group, *θ*_*π*_ = 1.539 and *θ*_*S*_ = 1.026 for the Central group and *θ*_*π*_ = 0.848 and *θ*_*S*_ = 1.283 for the Southern group (Table 1).

The hierarchical AMOVA analysis (Table 2) revealed clear structuring with high differentiation among groups (*Φ*_*CT*_ = 0.52, *Φ*_*ST*_ = 0.70, *p* < 0.001). The differences between the Northern and Central/Southern groups, Northern and Central groups and Northern and Southern groups were also high and statistically significant (*Φ*_*CT*_ = 0.45, *Φ*_*ST*_ = 0.75; *Φ*_*CT*_ = 0.43, *Φ*_*ST*_ = 0.70, *p* < 0.001 and *Φ*_*CT*_ = 0.65, *Φ*_*ST*_ = 0.80, *p* < 0.001, respectively), while the differences between the Central and Southern groups were moderate (*Φ*_*CT*_ = 0.48, *Φ*_*ST*_ = 0.63, *p* < 0.001, respectively).

**Table 2:** Hierarchical AMOVA analysis considering the three genetic groups suggested by the haplotype distribution.

Variation source	d.f.	Sum of squares	Variance component	% of variation
***Northern group vs. Central group vs. Southern group***				
Among groups	2	251.539	0.682	52.13
Among populations within groups	36	141.953	0.239	18.31
Within populations	540	208.820	0.387	29.56
Total	578	602.313	1.308	
*Φ*_*CT*_ = 0.52 (*p* < 0.001); *Φ*_*ST*_ = 0.70 (*p* < 0.001)				
***Northern group vs. Central-Southern groups***				
Among groups	1	124.357	0.703	45.25
Among populations within groups	37	269.135	0.464	29.86
Within population	540	208.820	0.387	24.90
Total	578	602.313	1.553	
*Φ*_*CT*_ = 0.45 (*p* < 0.001); *Φ*_*ST*_ = 0.75 (*p* < 0.001)				
***Northern group vs. Central group***				
Among groups	1	90.409	0.633	43.48
Among populations within groups	18	110.387	0.385	26.39
Within population	276	121.154	0.439	30.12
Total	295	321.949	1.457	
*Φ*_*CT*_ = 0.43 (*p* < 0.001); *Φ*_*ST*_ = 0.70 (*p* < 0.001)				
***Northern group vs. Southern group***				
Among groups	1	151.269	0.991	64.56
Among populations within groups	24	90.117	0.233	15.18
Within population	358	111.354	0.311	20.26
Total	383	352.740	1.535	
*Φ*_*CT*_ = 0.65 (*p* < 0.001); *Φ*_*ST*_ = 0.80 (*p* < 0.001)				
***Central group vs. Southern group***				
Among groups	1	127.182	0.539	48.44
Among populations within groups	30	83.402	0.158	14.24
Within population	446	185.133	0.415	37.32
Total	477	395.718	1.112	
*Φ*_*CT*_ = 0.48 (*p* < 0.001); *Φ*_*ST*_ = 0.63 (*p* < 0.001)				

*Φ*_*CT*_ = amount of differentiation among/between regions; *Φ*_*ST*_ = amount of differentiation among populations.

### Molecular signatures of demographic history

**Figure 3:**
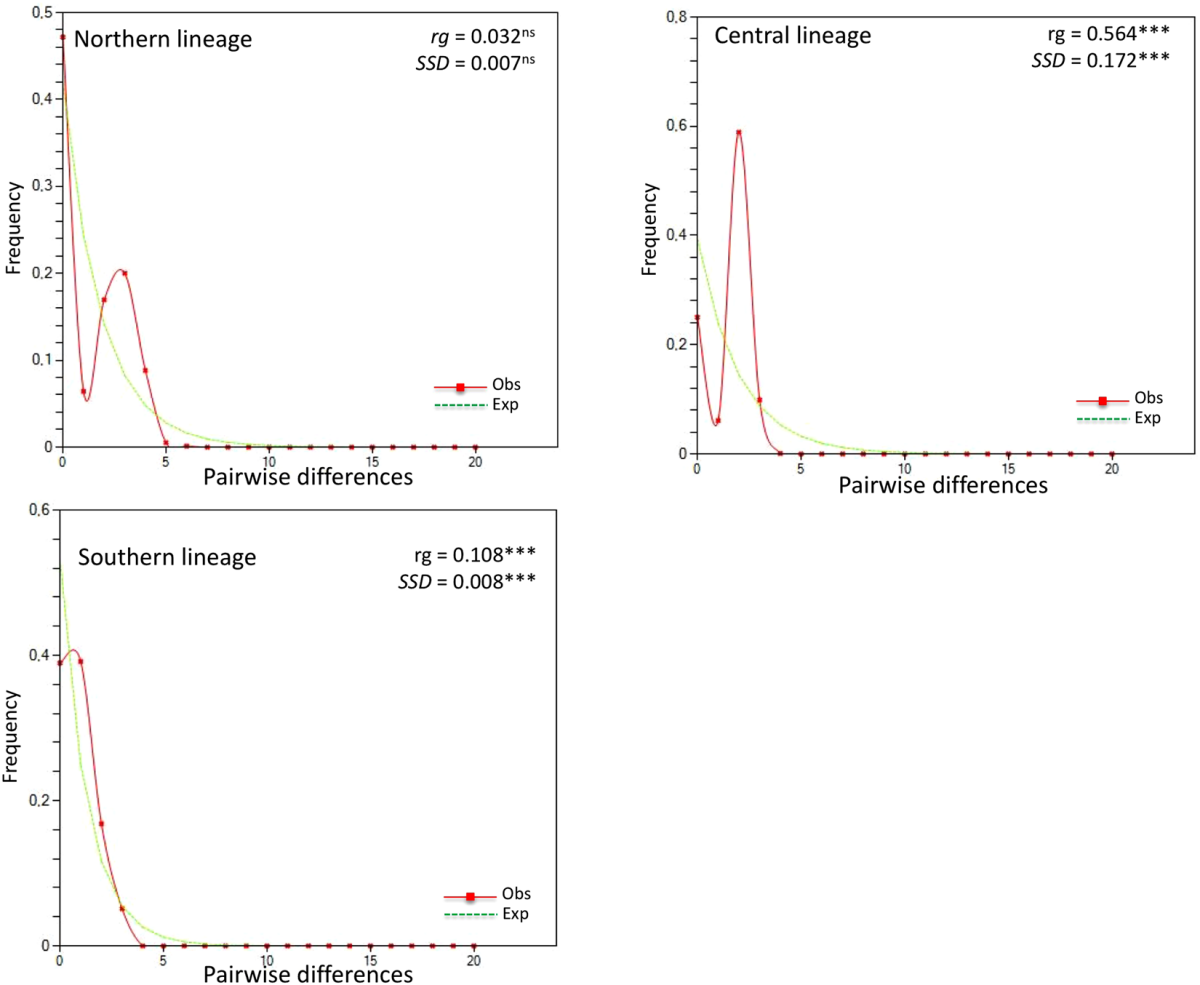
Mismatch distribution for the Northern, Central and Southern groups. Estimations of the raggedness (*rg*) and of the sum of squared deviations (*SSD*) index are presented with their respective significance, where ***p < 0.001 and ns = not significant.

Evaluating the haplotype network (Fig. 2) separately for each genetic group, star-like patterns are observed for Northern and Southern groups, suggesting that both experienced recent demographic expansions. Congruently, the mismatch distribution revealed a unimodal distribution of the pairwise differences for all genetic groups, suggesting population expansion (Fig. 3). However, the hypothesis test for population expansion through the *rg* and *SSD* statistics for the Northern group revealed low and statistically non-significant values (Fig. 3), not supporting population expansion for this particular group. On the other hand, the hypothesis of population expansion is validated for the Central and Southern groups through the statistically significant (*p* < 0.01) *rg* and *SSD* estimations (Fig. 3).

Tajima’s *D* and Fu´s *F*_*S*_ tests (Table 3) presented non-significant (*p* > 0.13) negative values for the Northern and Southern groups (*D*_(N)_ = −0.04, *F*_*S*(N)_ = −0.19 and *D*_(S)_ = −0.08, *F*_*S*(S)_ = −3.40) and positive values for the Central group (*D*_(C)_ = 1.02 and *F*_*S*(C)_ = 1.75).

**Table 3:** Estimations of demographic expansion parameters (Tajima’s *D*, Fu’s *F*_*S*_, mutational time since the expansion [*τ*] and initial [*θ*_0_] and final [*θ*_1_] theta) for each genetic group.

	Northern group (n = 101)	Central group (n = 195)	Southern group (n = 284)
*D* ^a^	−0.04 (*p* = 0.46)	1.02 (*p* = 0.87)	−0.08 (*p* = 0.19)
*F*_*S*_^a^	−0.19 (*p* = 0.51)	1.75 (*p* = 0.78)	−3.40 (*p* = 0.13)
*τ*	3.28 (1.25–6.86)	2.04 (0.75–3.31)	1.00 (0.87–1.39)
*θ*_*0*_^b^	0.00 (0.00–0.021)	0.00 (0.00–1.647)	0.00 (0.00–0.27)
*θ*_*1*_^b^	2.47 (1.90–20.59)	20.57 (3.40–271.82)	3407.18 (2.32–875.95)

^a^Estimations performed grouping all individuals of the whole range or of each genetic group as a single population.

^b^The confidence interval (5–95%) are given in parentheses.

The τ values (Table 3) suggest an older event of expansion for the Northern group (τ_(N)_ = 3.28), than for Central and Southern groups (τ_(C)_ = 2.04 and τ_(S)_ = 1.00). The similar estimations of *θ*_0_ for all genetic groups suggest a comparable intensity of one possible genetic bottleneck in the past (Table 3). As well, the high estimation of *θ*_1_ observed for the Southern group (Table 3) suggests the occurrence of bottleneck events. The large difference between *θ*_0_ and *θ*_1_ and the lack of superposition of the confidence intervals suggest the occurrence of population expansion for all genetic groups. Concerning the Central group, the τ estimation suggests an intermediary time of expansion, older then the Southern group and more recent then the Northern group, as previously suggested based on palynological and genetic data^19^.

**Figure 4:**
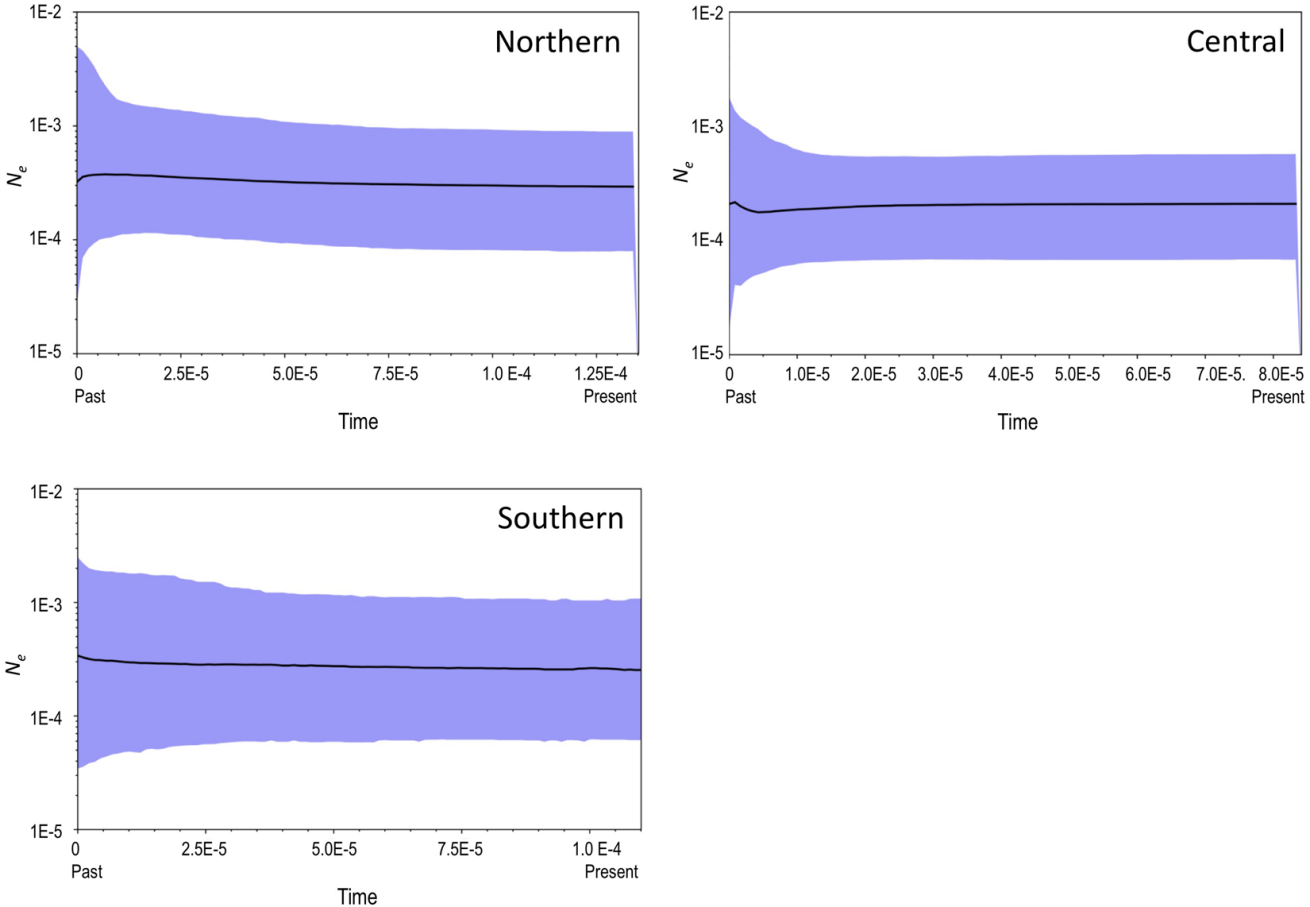
Bayesian Skyline plots derived from ptDNA sequences of *Araucaria angustifolia*. The analysis was performed for each of the determined genetic group. The thick solid line represents the mean effective population size *N*_*e*_, while the 95% HPD (highest posterior density) limits are shown by the blue area.

Opposing the mismatch distribution, *rg* and *SSD* statistics, the Bayesian skyline plot suggest that the median estimate of population size of all three genetic groups remained constant throughout the time (Fig. 4). Populations from the Northern genetic group revealed a quite small increase in the effective population size (*N*_*e*_) just after the coalescence. Contrarily, the Central genetic group experienced a small decline in *N*_*e*_ just after the coalescence, while no significant changes in *N*_*e*_ is observed in the Southern group.

### Paleodistribution and current distribution of *A. angustifolia*

**Figure 5:**
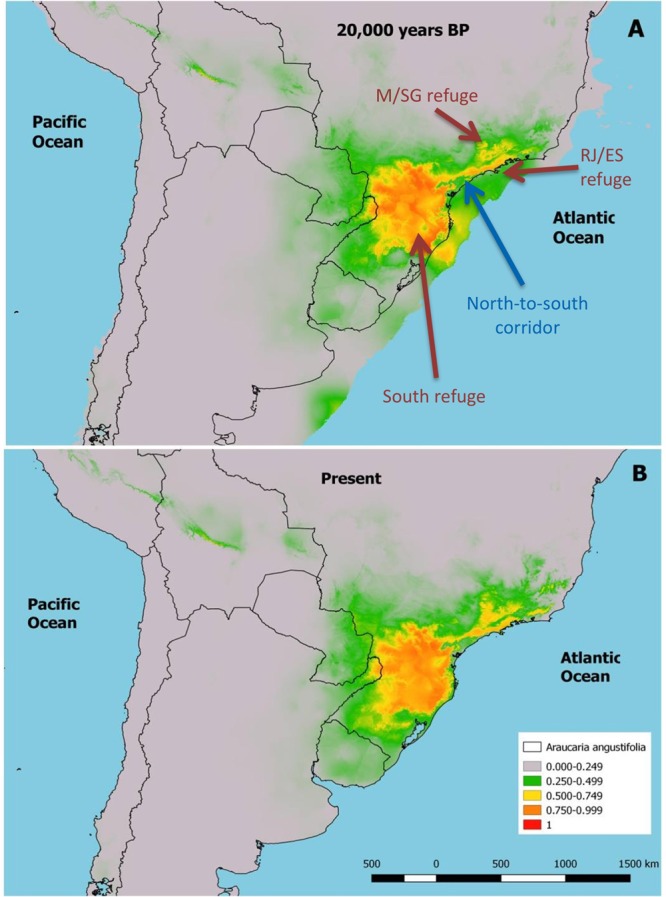
Species distribution modeling of *A. angustifolia*. (**A**) Predicted paleodistribution of *A. angustifolia* based on the climatic conditions of 20,000 years before present. The approximate location of the putative Rio de Janeiro/Espírito Santo (RJ/ES), Mantiqueira/Serra Geral (M/SG), and South refuges are indicated by red arrows. The blue arrow indicates the putative north-to-south corridor connecting the Northern and the Central regions. (**B**) Predicted occurrence based on current climatic conditions.

The predicted paleodistribution of *A. angustifolia* at 20,000 years before present (pROC = 0.998; Fig. 5A) and the predicted current distribution area (pROC = 0.990; Fig. 5B) have about the same pattern: a northernmost area that comprises part of the states of São Paulo, Minas Gerais and Rio de Janeiro, and a significantly larger southernmost area that comprises part of the states of Paraná, Santa Catarina and Rio Grande do Sul. The predicted current distribution matches the occurrence area of the species registered since the arrive of the Europeans in Brazil^15^, giving confidence for the ecological niche modelling.

The occurrence area predicted from the current climatic conditions is larger than the paleodistribution, but only slightly. The predicted paleodistribution suggests the presence of large areas of putative refugia with an occurrence probability of 100% for *A. angustifolia*, mainly in the southernmost range. In the northern range, almost all areas exhibit a probability of occurrence of 50% or less, restricting putative refugia areas during the glacial period. Probabilities of occurrence higher than 75% are observed only in small areas within the Mantiqueira/Serra Geral hills and in the coastal area where the occurrence of a glacial refuge has already been proposed^7,22^ (Fig. 5).

## Discussion

### The origin of recent populations of *A. angustifolia*: a mix of southward expansion and origin from multiple refugia

Our phylogeographic survey based on ptDNA sequences revealed genetic structuring across the full distribution range of *Araucaria angustifolia* (Fig. 1), with a marked north-to-south pattern. This phylogeographic structure supports the presence of three genetic groups proposed by the analysis of isozymes^9^ and nuclear SSR markers^10^. The phylogeographic structure revealed by several South American organisms (including algae, plants, birds, fishes and mammals) is typically stronger with uniparental than with biparental inherited markers^13^. *Araucaria angustifolia* revealed the same pattern in the analysis of uniparental ptDNA markers and biparental nuclear markers^10^, suggesting the correspondence of the current marker phylogeographic signature to the actual species phylogeography, with three main genetic groups. The origin of these three genetic groups, defined by the configurations of haplotype distribution, genetic diversity and genetic structure, can be explained by both the occurrence of southward expansion and the existence of further unidentified glacial refugia.

Late Quaternary range expansions in southern South America are seen in several plant (including trees, vines and bromeliads)^23–25^ and animal (including amphibians, squamates, birds and mammals)^26^ groups, suggesting that this might be a general pattern. During the Last Glacial Maximum, Australia and South America had their climates significantly modified, but were not subject to extensive glaciation^27^. Considering the substantial need for humidity by *A. angustifolia* (it needs a mean annual precipitation greater than 1,250 mm^28^), glacial refugia for this species in South America were related to localities with enough moisture for survival. Glacial refugia in Brazil have been proposed based on the distribution of woody angiosperm families^22^ and through species niche modeling of the Atlantic Forest^7^. Both strategies propose the existence of a refuge situated in the region of the states of Rio de Janeiro and Espírito Santo (RJ/ES refuge in Fig. 5A), where the Northern populations of *A. angustifolia* may have been originated. However, the genetic structure observed among the Northern populations, clearly revealing two main groups (Fig. 2 and Supplementary File 1) suggests the presence of a further northern refuge. As well, the area within the Matiqueira/Serra Geral hills (M/SG refuge in Fig. 5A) revealed by the niche modelling may be the origin of some Northern populations, since it has probability of species occurrence similar to the area of the already proposed RJ/ES refuge (Fig. 5A).

Likewise, populations from the Southern group can have been originated from a second refuge that coined haplotype H4 (see Fig. 5A), which is the overall most frequent haplotype that directly originated other six Southern haplotypes and is also found in three populations of the Central group (Fig. 2 and Supplementary File 1). Congruently, a small-scale phylogeographic study focused only on populations that represent the Southern genetic group^12^ reported a star-like haplotype network and negative estimations of Tajima’s *D* and Fu’s *F*_*S*_ neutrality tests, suggesting rapid and recent expansion of such populations from a single southern refuge.

The high and significant phylogeographic structure observed among the Northern, Central and Southern groups provides strong evidence on their origins from more than one glacial refugia^29^. Estimations of *Φ*_*CT*_ (differentiation between groups) and *Φ*_*ST*_ (differentiation among populations) are lower in the comparison between Central and Southern groups than estimations that include the Northern group (Northern *vs* Central/Southern, Northern *vs* Central and Northern *vs* Southern; Table 2), revealing the high divergence concerning this plastid group. In addition, the distribution modeling of *A. angustifolia* at 20,000 yBP reveals large areas with high probability for species occurrence within the southern distribution range, where glacial refugia could arise (South refuge, Fig. 5), as formerly proposed^8^.

The palynological record suggests that Northern populations of *A. angustifolia* expanded into highlands earlier than Central populations, while the Southern ones were the latest to experience demographic expansion^19^. Our estimations of the time of population expansion (*τ*) of the Central and Southern groups indicate a more recent origin in comparison to the Northern group (2.04 and 1.00 *vs* 3.28, respectively; Table 3), suggesting an earlier demographic expansion of Central and Southern populations, as proposed with base on palynological and nuclear genetic data^19^. Unlike the Northern populations that are isolated from the southernmost populations, the Central and Southern populations have a continuous distribution (Fig. 1), enabling gene flow that would explain the haplotypes shared between these two groups.

Even though the existence of a southernmost refuge cannot be discarded, the occurrence of southward expansion from a northern refuge is strongly evident and needed to explain the current distribution of *A. angustifolia*. Ancestral plastid haplotypes are likely to have given rise to a larger number of derived haplotypes than younger ones. Thus, ancestral haplotypes have more connections in a network because mutations have occurred over a longer period of time^30^, generating a star-like haplotype network. This is the pattern observed for haplotype H1, which is the common ancestor of the main haplotypes of the Central group. In addition, a close relationship between Northern and Central populations is observed in the Bayesian phylogenetic tree (Fig. 1) and in the SAMOVA analysis, since for some partition, one or two northern populations clustered with Central ones, but never with populations from the Southern group. Considering this scenario, populations of *A. angustifolia* may have expanded from north to south through an ancient corridor (Fig. 5A), originating some populations of the Central genetic group. These Central populations might further have mixed with Southern populations, originating the existing genetic structure.

The premise of the older origin of the Northern group is further supported by the measure of time since population expansion (*τ*, Table 3) and the expected pairwise differences before and after population expansion (*θ*_0_ and *θ*_1_, Table 3). Currently, the populations from the Northern group are geographically isolated from the southernmost ones, hindering gene flow between the Northern and the Central and Southern groups. Indeed, no shared haplotypes between populations of the Northern area and populations of the Central and Southern regions were observed. The ancient and contemporary presence of an area with low probability of species occurrence between the northernmost and the southernmost range of *A. angustifolia*, revealed by our modeling of the species distribution (Fig. 5), appears as an environmental barrier that isolates the Northern haplotypes. However, Central and Southern haplotypes are connected to the Northern haplotype H1 (Fig. 2), suggesting southward expansion throughout an ancient corridor that connected the northern and central distribution areas of *A. angustifolia* (Fig. 5A). In our modeling of species distribution for 20,000 yBP, a 50–75% probability of species occurrence was observed for this putative corridor towards the southwest (Fig. 5A).

The genetic diversity within and among populations is expected to be higher in refuge areas than in newly colonized regions^29^. In this study, the levels of *θ*_*π*_ (the average number of nucleotide differences per site between two sequences) declined in a north-to-south gradient (Table 1). The higher estimations of genetic diversity concerning this measure were observed for the Northern group, followed by the Central group, while the lowest estimations were found for the Southern group. The estimations of *θ*_*S*_ (the number of polymorphic sites) were higher for the Northern group, followed by the Southern and Central groups (Table 1). Altogether, *θ*_*π*_ and *θ*_*s*_ suggest that Central and Southern regions were more recently colonized, since genetic loci from a center of origin are expected to retain more ancestral variation and show higher nucleotide diversity, with group pruning through successive colonization events leading to a reduction in derived populations^31^.

### Demographic history of *A. angustifolia*

Negative values of Tajima’s *D* and Fu’s *F*_*S*_ statistics indicate an excess of rare alleles or new mutations in the genealogy that result from population expansion or genetic hitchhiking^32^. Northern and Southern genetic groups presented negative values for these statistics, but all statistically non-significant (Table 3). However, further important evidence about the demographic expansion experienced by *A. angustifolia* comes from the mismatch distribution and the Harpending’s raggedness and *SSD* analyses (Fig. 3). Populations undergoing demographic expansion usually present a unimodal mismatch distribution, while a multimodal distribution is expected for populations at demographic equilibrium, reflecting the stochasticity of gene trees. A unimodal mismatch distribution was observed for all genetic groups, but significant estimations of Harpending’s raggedness and *SSD* indexes were observed only for the Central and Southern groups.

These results contradict the Bayesian Skyline plot (BSP) analysis that revealed mainly constant size for all three genetic groups. These contrasting results may be effect of pooled sampling from a structured population that violates the assumption of panmixia in this analysis^33^ as well as in estimations of Tajima’s *D*^34^. It was shown that pooled sampling of structured populations with low geneflow generates a consistently higher inferred population size in the older parts of the Bayesian Skyline plots^33^. Therefore, the absence of signature of population growth in all three genetic groups of *A. angustifolia* might be an artefact of populations structure, increasing population size in the past area of the plots. Our pooled sampling (12–16 samples from 39 populations) is the most appropriate sampling strategy for analyzing structured populations^33^. Even though, the high mean *F*_*ST*_ estimations obtained from AMOVA analysis when the whole data is partitioned in three genetic groups (*F*_*ST*_ = 0.70; Table 2) suggests that the demographic history of *A. angustifolia* have to be interpreted in the proper context of its population structure.

Since population structure is a general problem that affects all methods that do not explicitly take subdivision into account (e.g. Tajima’s *D*, Fu’s *F*_*S*_ and BSP analyses), the demographic history of *A. angustifolia* have to be interpreted with clue off all pertinent approaches, including SAMOVA, AMOVA, *rg*, *SSD* and mismatch distribution analyses. Taking all analysis in account, we suggest that *A. angustifolia* genetic groups experienced fluctuations in effective size, including recent demographic expansions, also suggested in other studies^12,19^, allied to a structured distribution of populations.

### Highlights on species conservation

Besides assessing the current wide-scale genetic structure and diversity of tree species, it is also important to examine evolutionary and demographic histories of species, in order to understand the current distribution of genetic diversity, and to design long-term management and conservation programs^35^. The conservation of *A. angustifolia* seems to be directly related to the distribution of its genetic diversity, while the maintenance of current patterns of genetic variation depends on *in situ* conservation of the remnants and promotion of natural regeneration^10^. This strategy could be employed since the results of this study confirm the findings of previous investigations that found a strong genetic structure of *A. angustifolia* populations at different geographic levels^9,10,36^.

The present study confirms high differentiation regarding the Northern group compared to the Central and Southern ones and used a much larger and wider sampling strategy than previously reported. This differentiation is of great significance for the conservation of *A. angustifolia* genetic resources since it is possible that it reflects not only in the intergenic ptDNA regions investigated, as shown in this study, but also in genes of adaptive significance.

In addition to the glacial/post-glacial events, it is very likely that the shape of the genetic diversity and the current conservation status of *A. angustifolia* genetic resources were largely influenced by anthropic action^12,28^. While human populations may have partially promoted the dispersal of *A. angustifolia* seeds during migration events^12,20,28^, negative effects of forest fragmentation on the genetic diversity of populations were also described by Auler *et al*.^36^.

Conservation of forest genetic resources is directly associated to silvicultural management, genetic characterization and evaluation, nature conservation, legal regulations, political and socioeconomic issues, and overall development planning^37^. The use of geographic allocation of genetic variation as a pattern for planning the conservation of *A. angustifolia* has been proposed based on results of different studies^28^. The identification of three genetic groups in this study corroborates the proposition of using the geographic distribution of *A. angustifolia* as the principle way to select *in situ* conservation areas, for planning seed collection for *ex situ* conservation, and for the delineation of seed zones^10^.

## Material and Methods

### Sampling, DNA isolation and ptDNA sequencing

Sampling for this study included 580 adult individuals from 39 populations, distributed across most of the distribution range of the species, including Brazil and Argentina (Fig. 1). Leaf tissues from each tree were silica-dried and stored at room temperature until DNA extraction. Total DNA was isolated using the CTAB protocol, with modifications^38^. Three intragenic regions of the plastid genome of *A. angustifolia* were analyzed based on the polymorphism found by Schlögl *et al*.^39^ in a PCR-RFLP survey of natural populations of this species. The intragenic regions *trn*D-*trn*T (hereafter called DT), *psb*C-*trn*S (hereafter called CS) and *trn*S-*trn*fM (hereafter called SfM) were sequenced using universal primers^40^. Fragments were amplified using the following PCR conditions: 20 ng of template DNA, 0.2 μM of each primer, 2.5 mM of MgCl_2_, 1.0 U Taq polymerase, 1X PCR buffer, 0.3 mM of each dNTP, for a final volume of 20.0 μL. PCR cycles were performed as described by Demesure *et al*.^40^. PCR products were purified by precipitation using a 20% Polyethylene Glycol 8000 + 2.5 mM NaCl solution followed by washing in 80% ethanol and checking under UV in a 1% agarose gel stained with GelRed^®^ (Biotium^TM^). Sequencing reactions consisted of 1.0 μL of PCR product (100–200 ng μL^−1^), 0.35 μL of primer (10.0 μM) and 4.0 μL of DYEnamic^TM^ ET dye terminator^®^ mix (GE Healthcare, Little Chalfont, UK), for a final volume of 10 μL. Alternatively, some sequencing reactions were performed using 1.0 μL of PCR product (100–200 ng μL^−1^), 0.35 μL of primer (10.0 μM), 3.5 μL of 5X Sequencing Buffer and 1.0 μL of BigDye Terminator^TM^ v3.1 (Applied Biosystems, Carlbad, CA, USA), for a final volume of 20 μL. Each purified PCR product was subjected to DNA sequencing by capillary electrophoresis (CE) from both the forward and reverse directions using a MegaBACE 1000 DNA Sequencing System (GE Healthcare, discontinued in 2012), and an ABI3500XL Genetic Analyzer (Applied Biosystems), following the manufacturer’s instructions. Sequences were extracted using the programs Sequence Analyzer v. 4.0 (GE Healthcare) and Sequencing Analysis v5.4 (Applied Biosystems). Alignments and final concatenation were performed using the program CLC Genomics Workbench v.8 (Qiagen Bioinformatics, Hilden, Germany). DNA sequences were deposited in the NCBI database under ID numbers MH223675 – MH225411.

### Population structure and genetic diversity

Since the loci are linked in the plastid chromosome, all analyzes were performed with the concatenated sequences. All genetic parameters were estimated using the Tamura^41^ model of substitution, determined as the best model through the BIC scores (Bayesian Information Criterion) computed with the software Mega 6.0^42^. Evolutionary relationships among ptDNA sequences were evaluated by a median-joining^43^ using the software Network 5.0 (http://www.fluxus-engineering.com).

Previous AMOVA studies based on nuclear SSR and AFLP markers^10^ suggest the presence of three main genetic groups for populations of *A. angustifolia* across its distribution range in Brazil. A similar trend was observed in the distribution of plastid haplotypes (Supplementary File 1). In order to confirm this pattern, a spatial analysis of molecular variance (SAMOVA) was performed with SAMOVA v. 2.0^21^. This program finds the best number of geographic groups (*K*-value) by maximizing *F*_CT_ value between *K* groups of geographically adjacent populations. The number of geographic groups *K* was set from 2 to 15 and estimations were performed using the Tamura 2-parameters model.

Population genetic structure was then evaluated by means of a hierarchical AMOVA analysis considering the three genetic groups suggested by the haplotype distribution and SAMOVA analysis. A total of five AMOVA tests were performed: (i) Northern group *vs*. Central group *vs*. Southern group, (ii) Northern group *vs*. Southern and Central groups clustered, (iii) Central group *vs*. Southern group, (iv) Northern group *vs*. Central group, and (v) Northern group *vs*. Southern group. Computations were performed using the software Arlequin 3.5^44^.

Phylogenetic relationships among the 580 samples were estimated using a Bayesian Markov chain Monte Carlo approach as implemented in the software MrBayes 3.1^45^. A run length of 2.5 × 10^6^ generations was used under the HKY nucleotide substitution model, sample and print frequencies of 500, and diagnostic frequency of 5,000.

Patterns of genetic diversity were characterized by calculating (i) the total number of base mutations (SNPs) and indels, (ii) the total number of haplotypes, (iii) average number of pairwise differences *θ*_*π*_^46^ and (iv) nucleotide diversity *θ*_*S*_^47^. All estimations were calculated overall populations and for each defined genetic group (Northern, Central and Southern groups). Genetic groups were defined based on the haplotype distribution (see Fig. 2 and Supplementary File 1), SAMOVA analysis (Supplementary File 2) and previous evidence from isozyme and nuclear SSR analyses^9,10^. Computations were performed using the software Arlequin 3.5^44^.

### Estimation of demographic parameters

Based on the revealed genetic structure, evidence of demographic expansion/decline was assessed for each group (Northern, Central and Southern). Since contrasting results among different tests can occur (the *F*_*S*_ statistic is more powerful for capturing molecular signatures of population expansion, while Tajima’s *D* and the raggedness index are more conservative^48^), different tests were employed.

First, the mismatch distribution was computed using the software DnaSP 5.10^49^. In populations at demographic equilibrium, the distribution of pairwise differences usually presents a smooth decline, while it is usually unimodal and in the form of a wave in populations following a recent demographic expansion^50^.

The validity of the estimated demographic model was evaluated by computing the Harpending’s raggedness index^51^ (*rg*) and the sum of squared differences (*SSD*). Significance of *rg* and *SSD* were assessed through 1,000 bootstrap replicates. Statistically significant values were taken as evidence of departure from the null hypothesis of no ancient expansion. Computations were performed using Arlequin 3.5^44^.

Additionally, the hypothesis of population growth against the null hypothesis of a constant size population under the neutral model was tested using two complementary approaches: (i) Tajima’s *D*^46^, which is based on the difference between estimations of *θ*_*π*_ and *θ*_*S*_; and (ii) Fu’s *F*_*S*_^52^, which uses information from the haplotype distribution. Statistical significance of the analyses was determined through 10,000 simulations. The relative measure of time since population expansion in generations (*τ*) and the expected pairwise differences before and after change in population size (*θ*_0_ and *θ*_1_, respectively) were also estimated. All computations were performed using Arlequin 3.5^44^.

Further, the effective breeding population size (*N*_*e*_) through time was estimated for each genetic group using a non-parametric analysis based on the coalescence theory. The Bayesian skyline plot (BSP) approach was employed with the HKY substitution site model using a strict molecular clock with rate 1.0 and running 1.0 × 10^8^ Markov Chain Monte Carlo (MCMC) simulations in the software BEAST 2.0^53^. The convergence of the MCMC runs was evaluated by means of effective sample size (ESS) values for the parameters. Conditions of the MCMC runs were adjusted for reaching values ESS > 200 for all parameters (Supplementary File 4). The results were visualized by means of skyline plots using the software Tracer 1.6 (http://tree.bio.ed.ac.uk/software/tracer/).

### Modeling the paleodistribution and current distribution of *A. angustifolia*

Aiming to model the paleodistribution and current distribution of *A. angustifolia* populations, we employed the maximum entropy distribution model algorithm, using the software MaxEnt version 3.3.3^54^. This algorithm estimates the probability distribution for the occurrence of a species as a function of environmental restrictions. Based on species presence data and environmental variable layers of the study area, the model includes a deterministic outline, which enables performing confident analyses with information about presence-only point occurrences and high performance with few point localities^54,55^. The geographic data from the 39 sampled populations and additional points obtained from the literature were employed, which totaled 49 points of species occurrence distributed from 21°13′S to 30°30′S latitude and 43°46′W to 54°00′W longitude, covering the main distribution area of the species.

Nineteen bioclimatic variables available in the WorldClim database^56^ were extracted and used for the niche characterization in the modeling analysis. These metrics are derived from monthly temperature and rainfall values (11 temperature and eight precipitation metrics; Supplementary File 3), representing biologically meaningful variables for characterizing species distribution. Preliminary simulations were run excluding not correlated variables and the match of these simulations to the currently known area of species occurrence was used as criterium to determine the exclusion or not of such variables. The results of these simulations gave us confidence to perform the final analysis using all 19 variables. The geographic information system QGIS (http://qgis.org) was used to compile the MaxEnt results, interpolating climatic surfaces for global land areas in 2.5 arc minute maps (about 5 km^2^ resolution). Model validation was determined by the partial ROC (Receiver Operating Characteristics, pROC^57^) with values of pROC > 0.90 considered as excellent. A Jackknife test was employed to determine the prediction power of each variable by setting the variable aside from the test and generating its percentage of contribution to the model. All employed parameters were setting as follow: convergence threshold = 10^−5^; maximum iterations = 500; regularization multiplier = 1; application of a random seed, duplicate presence records removal and logistic probabilities used for the output. The model training was performed using 80% of species records and 20% was used to test the model.

In order to determine putative refugia for *A. angustifolia* populations at the end of the Last Glacial Maximum (20,000 years before present), the paleodistribution of the species was also predicted, using the bioclimatic variables from ECHAM3^58^. Data files were downloaded at their original 2.8125° resolution and resampled to 30” resolution via bilinear interpolation. Species paleodistribution modeling was conducted using the same parameters used for modeling current species distribution.

## Supplementary information

Supplementary Dataset 1
