# Supplementary material for: Phylogeography of plastid DNA sequences suggests post-glacial southward demographic expansion and the existence of several glacial refugia for Araucaria angustifolia
Source: Sci Rep. 2019 Feb 26;9:2752. doi: 10.1038/s41598-019-39308-w (PMC6391371; doi:10.1038/s41598-019-39308-w)
Supplement: Supplementary file 1 — Supplementary Dataset 1 [file 41598_2019_39308_MOESM1_ESM.pdf]

Phylogeography of plastid DNA sequences suggests post-glacial southward demographic expansion and the existence of several glacial refugia for *Araucaria angustifolia*

Valdir Marcos Stefenon, Gustavo Klabunde, Rafael Plá Matielo Lemos, Marcelo Rogalski, Rubens Onofre Nodari

Supplementary files

Table 1: Haplotype distribution in 39 populations of *A. angustifolia* sampled across the entire range of the species distribution.

File 2.1: Summary of the SAMOVA analysis with *K* set from 2 to 15.

File 2.2: Genetic structure suggested by the SAMOVA analysis for *K* = 3.

Table 3.1: Input file for the program MaxEnt, including the geographic coordinates off all populations sampled for this study.

Table 3.2: Percent contribution of the bioclimatic environmental variables for the predictive distribution of *A. angustifolia* in the maximum entropy analysis.

Table 4: Traces of the Bayesian Skyline analysis for each genetic group.

Supplementary Table 1: Haplotype distribution in 39 populations of *A. angustifolia* sampled across the entire range of the species distribution. The frequency of the haplotypes for each geographic-genetic group (N = Northern; C = Central; S = Southern) region is given. Haplotypes exclusive from the Northern group are in red, haplotypes exclusive from the Central group are in blue and haplotypes exclusive from the Southern group are in yellow. Haplotypes shared between Northern and Southern populations are in green.

	Haplotype																			
	H1	H2	H3	H10	H17	H18	H19	H20	H16	H15	H4	H9	H11	H12	H5	H6	H7	H8	H13	H14
Region																				
N	1.0	1.0	1.0	1.0	1.0	1.0	1.0	1.0	0.0	0.0	0.0	0.0	0.0	0.0	0.0	0.0	0.0	0.0	0.0	0.0
C	0.0	0.0	0.0	0.0	0.0	0.0	0.0	0.0	1.0	0.92	0.06	0.96	0.03	0.89	0.0	0.0	0.0	0.0	0.0	0.0
S	0.0	0.0	0.0	0.0	0.0	0.0	0.0	0.0	0.0	0.08	0.94	0.04	0.97	0.11	1.0	1.0	1.0	1.0	1.0	1.0
Pop																				
BAR	8	6	1																	
CDJ	2		7	6																
IBI					9	5														
MVE					9	6														
PES					13	2	1													
SAB					10			2												
VMA					12	2														
MRV										8	4	3								
CWB										7		7		1						
FXN										3		6		6						
IMB												5		10						
IRT										1		13		1						
LJS									3	7				5						
MRN									2			7		6						
PGR									2			11		2						
TRV									1					14						
PLM									3	7				5						
CHP											7			8						
DSV									5				1	9						
PBC										3	8			4						
MIS													8	3			3			1
PTS											7		7	1						
CCD											10	1					4			
VDR											7	1						7		
BJS											9				5	1				
BRT											11						4			

CBS	9						5	1		
CDR	9		6							
CNS	6							8	1	
CPV	5		1			7				1
CTV	9						6			
GMD	10					4				1
LGS	7		6				2			
LVM	11		1				3			
NPT	10					3				2
PFD	12		2						1	
RQM	13		2							
SJQ	4		5		1		3	2		
URU	14						1			

Supplementary File 2.1: Summary of the SAMOVA analysis with *K* set from 2 to 15.

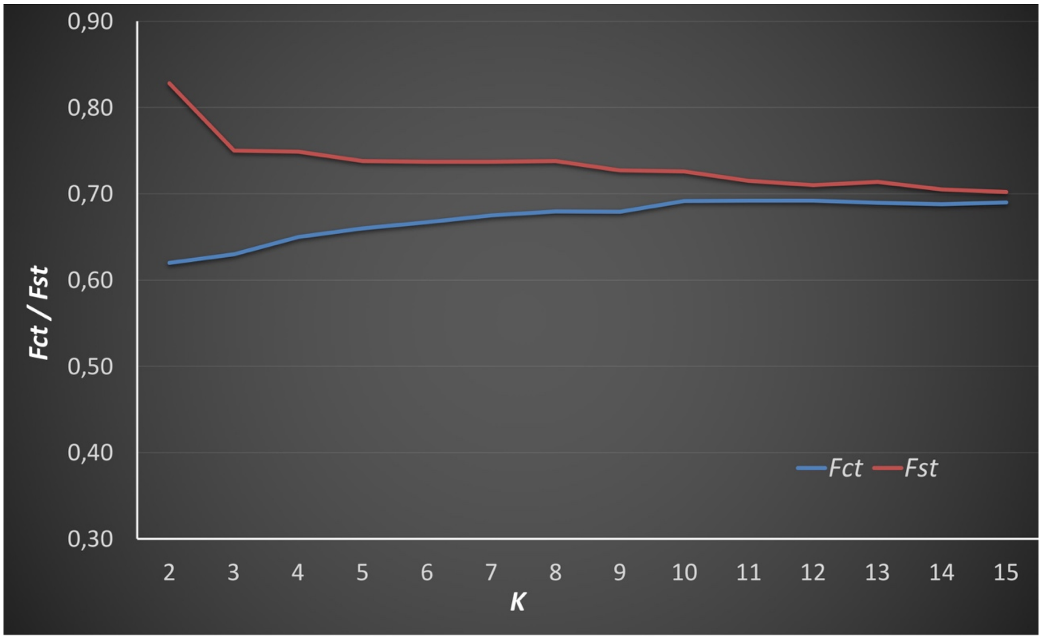

Supplementary File 2.2: Genetic structure suggested by the SAMOVA analysis for *K* = 3.

```
---------------------------
Genetic structure to test:
---------------------------

No. of Groups = 3

        [[Structure]]

                StructureName = "3 groups of populations"
                NbGroups = 3

        #Group1
                Group={
                "BAR"
                "CDJ"
                "MRV"
                "CWB"
                "FXN"
                "IMB"
                "IRT"
                "LJS"
                "MRN"
                "PGR"
                "TRV"
                "PLM"
                "CHP"
                "DSV"
```

```
        }

#Group2
        Group={
        "IBI"
        "MVE"
        "PES"
        "SBA"
        "VMA"
        }

#Group3
        Group={
        "PBC"
        "MIS"
        "PTS"
        "CCD"
        "VDR"
        "BJS"
        "BRT"
        "CBS"
        "CDR"
        "CNS"
        "CPV"
        "CTV"
        "GMD"
        "LGS"
        "LVM"
        "NPT"
        "PFD"
        "RQM"
        "SJQ"
        "URU"
        }
```

Supplementary Table 3.1: Input file for the program MaxEnt, including the geographic coordinates off all populations sampled for this study.

species	dd long	dd lat
Araucaria	-44.4335	-22.0215
Araucaria	-45.1325	-21.1154
Araucaria	-46.1637	-22.4846
Araucaria	-44.3607	-22.1922
Araucaria	-44.5432	-21.5903
Araucaria	-43.5432	-21.4352
Araucaria	-50.39	-28.32
Araucaria	-50.25	-27.54
Araucaria	-49.59	-28.12
Araucaria	-49.26	-27.47
Araucaria	-49.09	-27.39
Araucaria	-51.00	-26.5
Araucaria	-51.05	-27.22
Araucaria	-51.55	-27.21
Araucaria	-49.59	-26.23
Araucaria	-50.37	-27.18
Araucaria	-51.07	-27.01
Araucaria	-52.19	-28.14
Araucaria	-51.39	-27.03
Araucaria	-50.59	-29.21
Araucaria	-51.33	-28.12
Araucaria	-51.50	-26.54
Araucaria	-49.19	-25.25
Araucaria	-50.52	-25.22
Araucaria	-52.28	-26.22
Araucaria	-50.18	-25.02
Araucaria	-51.32	-25.03
Araucaria	-52.23	-25.22
Araucaria	-52.53	-25.55
Araucaria	-50.45	-24.27
Araucaria	-52.50	-25.44
Araucaria	-51.40	-24.53
Araucaria	-51.19	-23.59
Araucaria	-51.39	-24.24
Araucaria	-45.35	-22.44
Araucaria	-54.00	-26.51
Araucaria	-53.29	-30.30
Araucaria	-49.35	-28.00
Araucaria	-43.46	-21.13
Araucaria	-51.07	-29.23

Supplementary Table 3.2: Percent contribution of the bioclimatic environmental variables for the predictive distribution of *A. angustifolia* in the maximum entropy analysis.

BioClim Legend	Environmental Variables	Percent of contribution
BIO 17	Precipitation of Driest Quarter	27.4
BIO 18	Precipitation of Warmest Quarter	20.7
BIO 3	Isothermality (BIO2/BIO7) × (100)	20.5
BIO 4	Temperature Seasonality (standard deviation × 100)	14.1
BIO 11	Mean Temperature of Coldest Quarter	8.1
BIO 14	Precipitation of Driest Month	4.6
BIO 10	Mean Temperature of Warmest Quarter	1.7
BIO 1	Annual Mean Temperature	0.0
BIO 2	Mean Diurnal Range [Mean of monthly (max temp-min temp)]	0.0
BIO 5	Max Temperature of Warmest Month	0.0
BIO 6	Min Temperature of Coldest Month	0.0
BIO 7	Temperature Annual Range (BIO5 – BIO6)	0.0
BIO 8	Mean Temperature of Wettest Quarter	0.0
BIO 9	Mean Temperature of Driest Quarter	0.0
BIO 12	Annual Precipitation	0.0
BIO 13	Precipitation of Wettest Month	0.0
BIO 15	Precipitation Seasonality (coefficient of variation)	0.0
BIO 16	Precipitation of Wettest Quarter	0.0
BIO 19	Precipitation of Coldest Quarter	0.0

Supplementary Table 4: Traces of the Bayesian Skyline analysis for each genetic group. ESS (effective sample size) is an estimate of how many effectively independent draws from the marginal posterior distribution the MCMC is equivalent to. ESS > 200 assures that the MCMC simulation provides a very good estimate of the posterior distribution.

	Northern genetic group		Central genetic group		Southern genetic group	
Statistic	Mean	ESS	Mean	ESS	Mean	ESS
Posterior	-2588.928	1341	-1768.014	436	-1823.179	542
Likelihood	-3340.138	2774	-3319.100	1700	-3369.527	1628
Prior	751.210	1376	1551.086	439	1546.348	546
treeLikelihood	-3340.138	2774	-3319.100	1700	-3369.527	1628
TreeHeight	5.603E-4	8962	4.127E-4	6225	4.791E-4	1924
gammaShape	1.027	3109	0.985	2690	0.712	2174
BayesianSkyline	718.525	1376	1515.998	441	1513.925	542
bPopSizes. 1	7.473E-4	1277	5.790E-4	329	7.694E-4	296
bPopSizes. 2	4.582E-4	2029	2.806E-4	645	3.799E-4	891
bPopSizes. 3	3.950E-4	3414	2.047E-4	1634	3.917E-4	1462
bPopSizes. 4	3.680E-4	7829	1.996E-4	2585	4.705E-4	2124
bPopSizes. 5	3.148E-4	6630	2.372E-4	4180	3.484E-4	1313
bGroupSizes. 1	19.440	8237	37.227	1610	43.134	1502
pGroupSizes. 2	19.848	16038	38.651	2899	45.304	2744
pGroupSizes. 3	20.068	18899	39.708	3360	45.557	2279
pGroupSizes. 4	20.181	18163	40.607	2168	42.947	2625
pGroupSizes. 5	20.463	11422	37.807	1785	35.059	1688
kappa	1.215	3836	1.791	3275	1.87	5090
freqParameter. 1	0.284	4941	0.285	5464	0.285	4669
freqParameter. 2	0.180	5349	0.181	5708	0.182	5161
freqParameter. 3	0.218	5078	0.217	5749	0.217	4733
freqParameter. 4	0.318	4758	0.317	5603	0.316	4731
